# Cue-Reminders to Prevent Health-Risk Behaviors: A Systematic Review

**DOI:** 10.3389/fpubh.2019.00097

**Published:** 2019-04-30

**Authors:** Lonneke van Leeuwen, Simone Onrust, Bas van den Putte, Marloes Kleinjan, Lex Lemmers, Rutger C. M. E. Engels, Roel C. J. Hermans

**Affiliations:** ^1^Trimbos Institute, Netherlands Institute of Mental Health and Addiction, Utrecht, Netherlands; ^2^Amsterdam School of Communication Research, University of Amsterdam, Amsterdam, Netherlands; ^3^Department of Interdisciplinary Social Sciences, Utrecht University, Utrecht, Netherlands; ^4^Erasmus School of Social and Behavioural Sciences, Erasmus University, Rotterdam, Netherlands; ^5^Behavioural Science Institute, Radboud University, Nijmegen, Netherlands; ^6^Department of Health Promotion, NUTRIM School of Nutrition and Translational Research in Metabolism, Maastricht University, Maastricht, Netherlands

**Keywords:** cue-reminder, reminder cue, health promotion, health-risk behaviors, intervention programs

## Abstract

**Introduction:** It has been proposed that the use of cue-reminders may increase the effectiveness of interventions that aim to prevent health-risk behaviors (i.e., having unsafe sex, unhealthy dietary intake, lack of physical activity, and substance use). The aim of this systematic review was to explore whether there is evidence supporting this proposition, and to explore how cue-reminders are applied in health-risk behavior interventions to date.

**Method:** We systemically reviewed (non-) randomized trials that examine differences in health-risk behaviors between an experimental group receiving an intervention with exposure to a cue-reminder and a control group receiving the intervention without such cue.

**Results:** Six studies were eligible for inclusion. The studies differed in sample and research design, and how the cue-reminder was applied. One study demonstrated a positive and small effect, and one study found a negative medium effect of the cue-reminder. In the remaining studies, the effect sizes were positive but non-significant.

**Discussion:** It is unclear whether complementing health-risk behavior interventions with cue-reminders increases the effectiveness of these interventions. Further investigation and experimentation into the efficiency and effectiveness of cue-reminders is needed before health-risk behavior interventions are complemented with cue-reminders.

In Western society, the most prominent contributors to mortality and morbidity can be linked to health-risk behaviors, such as having unsafe sex, unhealthy dietary intake, lack of physical activity, and substance use (1). Consequently, many health-promoting organizations develop and implement interventions to prevent or reduce these health-risk behaviors. Despite the aim of these interventions to help people maintain and improve their health, evidence indicates that post-intervention changes are difficult to sustain (2). Merely through the passing of time, intervention recipients tend to forget what was learned during interventions (3) and easily return to their initial behavior(s) once they face the tempting daily life situations in which they used to enact these health-risk behaviors (2).

To increase the effectiveness of health-risk behavior interventions (hereafter: interventions), it is proposed that offering intervention recipients a cue-reminder may be an effective strategy (2, 4). Cue-reminders are grouped in the “associations” category of the Behavior Change Taxonomy (5). A cue-reminder is an object that is aimed to increase the salience of an intervention message at the time and place where normally the health-risk behavior would occur. The cue-reminder is introduced during a learning situation (the intervention), thereby aiming to create an association between the cue-reminder and the learning situation (3). Observing this cue-reminder in potentially risk behavior-inducing contexts may facilitate the reactivation and retrieval of relevant memories associated with intervention (3), such as the experience of having received the intervention or the specific health information received during the intervention. Further, cue-reminders may serve as a reminder of intentions, thereby supporting intervention recipients in turning their intentions into behaviors (6). Lastly, cue-reminders may help to inhibit social reactive processes that could negatively influence recipients' behaviors, such as conforming to pro-alcohol social norms (7, 8). It has been found that inhibitory cues, when made salient, impede impulsive behaviors, and counteract seducing or appealing cues or pressures present in that specific situation (4, 9). Thus, to summarize, cue-reminders may help to increase the salience of a risk-behavior inducing situation and reinforce actions needed to avoid this situation and adapt one's behavior accordingly to the intervention goal. An example of a cue-reminder in a health-risk behavior intervention is a bracelet worn by intervention recipients, as a reminder of the dangers of unsafe sex and to encourage condom use during intercourse (4).

Although complementing interventions with cue-reminders may be intuitively appealing, little is known about the effectiveness of cue-reminders in the context of health-risk behaviors (i.e., having unsafe sex, unhealthy dietary intake, lack of physical activity, and substance use), and how cue-reminders are best be applied. Therefore, the first objective of this systematic review is to explore whether there is evidence supporting the proposition that complementing health-risk behavior interventions with cue-reminders increases the effectiveness of such interventions. The second objective is to explore how cue-reminders are applied, because this may inform risk-behavior preventing organizations who are planning to complement their interventions with cue-reminders. To reach these objectives, we systematically review (non-) randomized trials that examine differences in health-risk behaviors between an experimental group receiving an intervention with exposure to a cue-reminder and a control group receiving the intervention without such cue.

## Methods

This systematic review was prepared in accordance with the PRISMA statement for the reporting of systematic reviews (10). A review protocol was established in preparation of this systematic review (not registered).

### Selection of Studies

#### Eligibility Criteria

There were six inclusion criteria. First, eligible studies should be randomized controlled trials or non-randomized controlled trials. Second, the experimental condition should involve exposure to an intervention followed by exposure to a cue-reminder. The control/comparison condition should consist of exposure to the same intervention but without exposure to the cue-reminder. We conceptualized cue-reminders as objects that provide reminder cues or subtle stimuli to intervention recipients with the aim to activate and retrieve intervention-related memories. Therefore, studies that did not involve an object as a reminder were to be excluded. Third, eligible studies should assess the impact of interventions on behavioral outcomes related to one or more of the following health-risk behaviors: having unsafe sex, unhealthy dietary intake, lack of physical activity, and substance use (i.e., tobacco, alcohol, illicit drugs). Fourth, eligible studies should report results about the added effect of a cue-reminder on behavioral outcomes or data from which this added effect can be calculated. Fifth, eligible studies should be written in English. Sixth, study subjects should be human.

#### Search Strategy

Four electronic databases (PsycInfo, PubMed, CINAHL, and EMBASE) were searched for eligible studies (search date: July 10th 2017). The search strategy is described in Table 1. No restrictions were imposed on studies' publishing dates or sample characteristics. Studies listed in these databases were retrieved by using combinations of thesaurus terms, key words, and text words in titles and abstracts. These terms and words refer to health-risk behaviors, cue-reminders, and interventions. In total 2,101 records were retrieved: 389 records were retrieved by the search in PsycInfo, 579 by PubMed, 197 by CINAHL, and 936 by EMBASE. All records were combined in Reference Manager Version 12 (Thomson Reuters, Philadelphia, PA, USA). Removal of duplicates resulted in 1,397 unique records. In addition, the reference lists of eligible studies were hand searched to identify additional eligible studies, as well as the studies that cited eligible studies. This resulted in the identification of 17 additional potential eligible studies.

**Table 1:** Applied search strategy.

**Database**	**Strategy**	**Search terms**
PsycInfo	Thesaurus terms referring to health-risk behaviors and their healthy counterparts	Safe sex, AIDS prevention, active living, diets, exercise, physical activity, weight control, condoms, overweight, obesity, food intake, eating behavior, smoking cessation, tobacco smoking, alcohol drinking patterns, binge drinking, drug abuse, drug usage, alcohol abuse, drug abuse prevention
	Thesaurus terms, keywords, and words in title or abstract referring to cue-reminders	Thesaurus terms: cued recall, cues Keywords or words in title or abstract: cue-reminder^*^, cued behavior^*^, cued behavior^*^, cued behavior^*^, reminder cue^*^, cue based intervention^*^, cue to act, external cue^*^, reminder^*^, external memory aid^*^, explicit cue^*^, cueing, cue to action^*^, cues to action^*^
	Keywords and words in title or abstract referring to interventions	Intervention^*^, program^*^
PubMed	Thesaurus terms related to health-risk behaviors and their healthy counterparts	Smoking, marijuana smoking, life style, binge drinking, alcohol drinking, drinking behavior, substance-related disorders, alcoholism, marijuana abuse, opioid-related disorders, obesity, diet, tobacco use cessation, smoking cessation, exercise, weight loss, overweight, diet-reducing, feeding behavior, condoms/utilization, safe sex
	Thesaurus terms and words in title or abstract referring to cue-reminders	Thesaurus terms: Reminder systems, cues Words in title or abstract: cue-reminder, cue-reminders, cued behavior^*^, cued behavior^*^, reminder cue^*^, cue based intervention^*^, cue to act, cues to act, external cue^*^, reminder^*^, external memory aid^*^, explicit cue^*^, cueing, cue to action^*^, cues to action^*^
	Words in title or abstract referring to interventions	Intervention^*^, program^*^
CINAHL	Thesaurus terms referring to health-risk behaviors and their healthy counterparts	Smoking cessation, smoking cessation programs, smoking, substance dependence, substance use disorders, substance abuse, alcohol-related disorders, alcohol abuse, alcoholism, binge drinking, condoms+, diet, exercise, food intake, obesity, physical activity, safe sex, weight control, weight loss, weight reduction programs, alcohol drinking, binge drinking, drinking behavior, eating behavior+
	Thesaurus terms, and words in title or in abstract referring to cue-reminders	Thesaurus terms: reminder systems, cues. Words in title or in abstract: Cue-reminder^*^, cued behavior^*^, cued behavior^*^, cued behavior^*^, reminder cue^*^, cue based intervention, cue to act^*^, cues to act^*^, external cue^*^, reminder^*^, external memory aid^*^, explicit cue^*^, cueing, cue to action^*^, cues to action^*^
	Words in title or abstract referring to interventions	Intervention^*^, program^*^
EMBASE	Thesaurus terms referring to health-risk behaviors and their healthy counterparts	Drug abuse, alcoholism, drug dependence, tobacco dependence, condom, obesity, weight reduction, diet, feeding behavior, exercise, drinking behavior, smoking cessation
	Thesaurus terms, and words in title or in abstract referring to cue-reminders	Thesaurus term: reminder system, association Words in title or in abstract: Cue reminder^*^, cued behavior^*^, cued behavior^*^, cued behavior^*^, reminder cue^*^, cue based intervention^*^, cue to act^*^, cues to act^*^, external cue^*^, reminder^*^, external memory aid^*^, explicit cue^*^, cueing, cue to action^*^, cues to action^*^
	Thesaurus term, and words in title or abstract referring to interventions	Thesaurus term: intervention study Intervention^*^, program^*^

Potential eligible but unpublished studies were searched by a variety of strategies. First, the authors shared an online request for unpublished studies through email and (professional) networking accounts amongst peer researchers and health promoters. Second, authors from eligible studies as well as authors who cited eligible studies were contacted by email with this request. Third, a request for unpublished studies was posted on the forum of the *Society for Personality and Social Psychology*. Nine researchers responded with suggestions for unpublished and potentially eligible studies, resulting in the identification of six additional potentially eligible studies. In total, the search strategy for this systematic review yielded 1,420 potentially eligible records.

**Figure 1:**
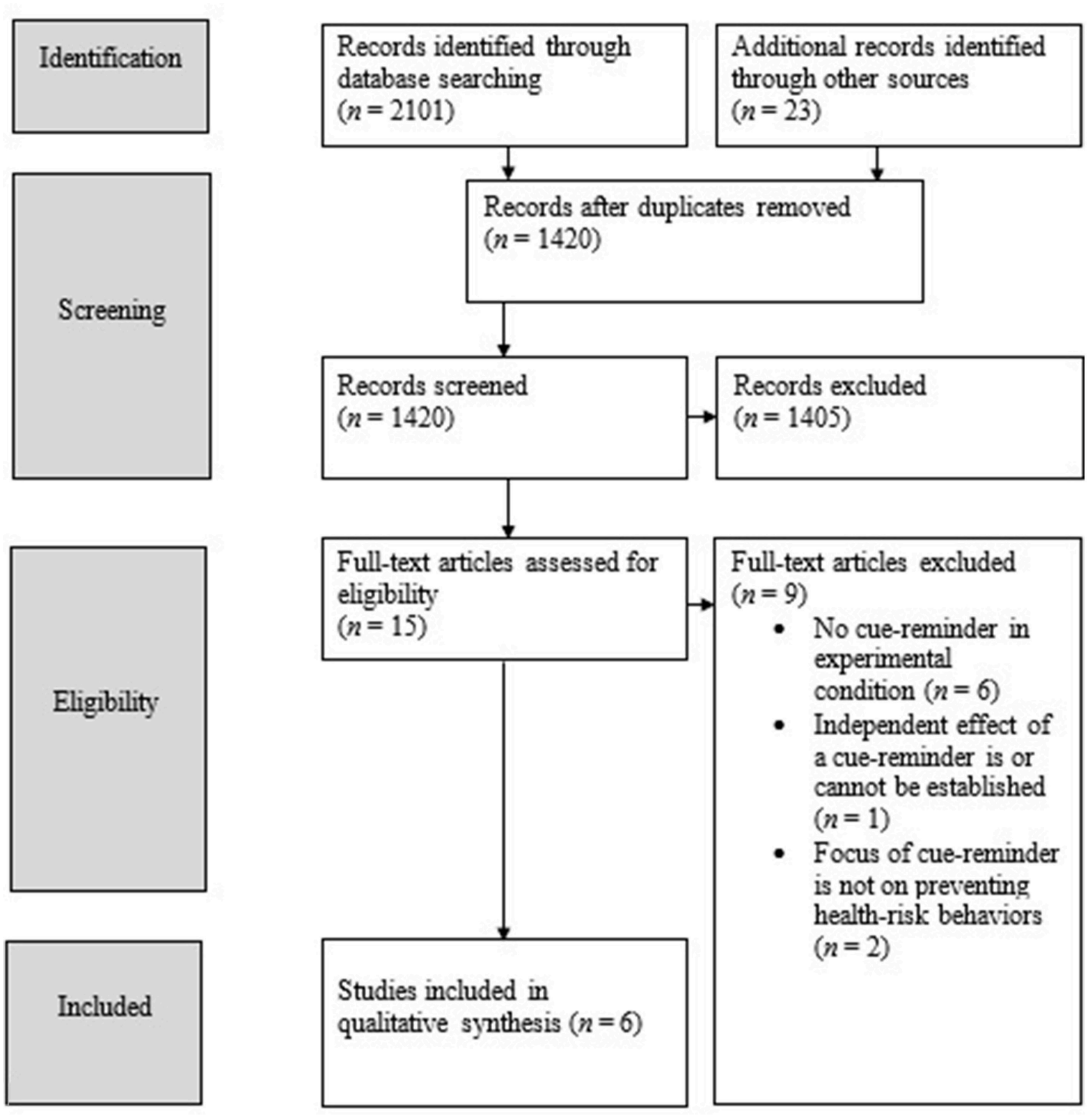
Overview of screening and selection procedures.

The titles and/or abstracts of the 1,420 identified records were then screened by two researchers (by LL/RCJH and LvL) to assess whether the studies met the inclusion criteria. In cases of disagreement, titles or abstracts were discussed until consensus about potential eligibility was achieved. Based on this screening, 1,405 records were excluded. The majority of records were excluded because the studies did not investigate cue-reminders but focused on associative, situational, or environmental cues, such as images of alcoholic drinks or food. Thus, these studies focused on cue-reactivity in health-risk behavior rather than on the use of cue-reminders to prevent those behaviors. Also, in many studies, the abstract showed that more differences existed between the experimental and control conditions than the presence/absence of cue-reminders only. For example, there were studies in which the experimental group received an intervention including a cue-reminder, but in which the control group received no intervention at all. Next, the full-text articles of the remaining 15 records were retrieved and screened against the inclusion criteria (by LvL and RCJH). In the case of disagreement, studies were discussed until consensus about eligibility was achieved. The full-text screening resulted in the exclusion of another nine articles for three reasons. Firstly, the full-text versions showed that the reminder was not an object but, for example, a text message. Secondly, the full-text versions indicated more differences between the experimental and control conditions than the presence/absence of cue-reminders alone. Thirdly, full-text versions indicated that the focus of the reminder was not on one of the four health-risk behaviors selected for this review. The remaining six studies were included (see Figure 1 for the overview of screening and selection procedures).

### Data Extraction and Risk of Bias

The following data were extracted from each study: outcome measure(s), sample size, participants' mean age, sample size per condition, number and timing of measurements, information about the content of the intervention, and information about the design and implementation of the cue-reminder. To assess the risk of bias of included studies, two researchers (SO and LvL) independently completed the risk of bias assessment tool of The Cochrane Collaboration (11). After completion, the researchers discussed any disagreement until consensus was achieved. The results of the risk of bias assessment were used to describe the overall risk of bias across the included studies.

### Data Analysis

Effect sizes were calculated for each comparison of the impact of an intervention plus cue-reminder condition vs. an intervention only condition. The calculated effect sizes were standardized mean differences (Cohen's *d*).

Effect sizes per outcome per study were calculated with the software program Comprehensive Meta-Analysis (CMA; version 2.0; Biostat, Englewood, New Jersey). For the calculation of effect sizes for which no procedures were available in CMA, an online effect size calculator was used (12). In cases where multiple outcome measures were used to assess the same behavior, for example quantity and frequency of alcohol consumption, the effects sizes per outcome measure were pooled to yield a single effect size. Positive effect sizes indicate a positive added effect of the cue-reminder, such that participants in the intervention plus cue-reminder condition showed less health-risk behavior than participants in the intervention only condition. Following Lipsey (13), Cohen's *d*s of < 0.32 can be considered as small effect sizes, between 0.32 and 0.55 as medium effect sizes, and larger than 0.55 as large effects sizes.

## Results

### Overview

Detailed information of each included study is shown in Table 2. Of the six studies, four studies focused on substance use (alcohol consumption) (14, 16–18), one study focused on unhealthy dietary intake (i.e., candy consumption) (15), and one study focused on unsafe sex (4). The cue-reminders that complemented the interventions were a bracelet (4, 17), a bracelet or a self-selected cue (14), a monkey puppet (15), and drink coasters (16, 18).

**Table 2:** Descriptive information and effect sizes of the included studies.

**1st-named author, year**	**Outcome measure(s)**	***N***	**Participants' mean age (*SD*)**	**Conditions (C) and (sample size)**	**Measurements**	**Description of intervention**	**Description of the cue-reminder**	**Characteristics of cue-reminder design and implementation**	**Effect size per study (95% CI) and significance**
Van Lettow (14)	Self-report of alcohol consumption (quantity and frequency)	2,634	37.0 (15.2)	C1: intervention only (*n* = 1,520)C2: intervention plus cue-reminder (*n* = 1,113)	T1: pre intervention T2: 1 month after intervention (and after cue-reminder exposure in C2)	Completion of an online feedback program (Drinktest.nl). Reception of individually tailored messages aimed at reducing alcohol drinking. Feedback on prototype characteristics. Action planning.	Wearing an offered bracelet or self-selected cue. Exposure during ≥1 month after intervention	Explicit instruction about cue-reminder aim.Duration of exposure: 1 month.	+0.17 (0.09 to 0.24), *p* = 0.00
Bevelander (15)	Objectively assessed candy consumption (consumed kilocalories)	141	7.84 (0.72)	C1: intervention only (*n* = 43)C2: intervention plus cue-reminder (*n* = 43)	T1: directly after intervention (and after cue-reminder exposure in C2)	An 8-min session for groups of 5–7 participants in a classroom. In a short interactive lesson featuring photos, video clips, and real-life situations, the children were taught the meaning of peer modeling and why it is healthier to pay attention to how much they eat in the company of their peers.	Exposure to monkey puppet The monkey puppet was present during a 10-min test session in which the participants were allowed to eat candy	No explicit instruction about cue-reminder aim.Duration of exposure: 10 min	+0.32 (−0.10 to 0.75), *p* = 0.14
Kleinjan (study 2) (16)	Objectively assessed alcohol consumption (alcohol vs. no alcohol consumed, number of alcoholic consumptions)	107	20.9 (1.77)	C1: intervention only (*n* = 54)C2: intervention plus cue-reminder (*n* = 53)	T1: directly after intervention (and after cue-reminder exposure in C2)	Exposure to a film about environmental pressure and alcohol use. It focused on social norms, pressure to drink, external influences of commercials, and happy hours and refusal skills. A symbol resembling a power button was embedded in the film.	Exposure to double-sided coasters with pictures of the symbol (power button). The coasters were added to 45-min visit to an experimental drinking situation where they were distributed on all the tables in the bar.	No explicit instruction about cue-reminder aim.Duration of exposure: 45 min	+0.17 (−0.26 to 0.59), *p* = 0.44
Dal Cin (4)	Self-report of condom use (mean % condom use, % condom use at last intercourse)	196	19.58 (1.77)	C1: intervention only (*n* = 47)C2: intervention plus cue-reminder (*n* = 42)	T1: 5–7 weeks after intervention (and after cue-reminder exposure in C2)	In a classroom setting, the viewing of a documentary in which young people living with HIV/AIDS describe their experiences, including contracting the virus through unprotected sexual intercourse.	Wearing of a bracelet. Participants were told to remember the stories of the people in the video whenever they looked at the bracelet to remind them of the dangers of unsafe sex. Participants were requested to wear the bracelet at all times until the posttest measurement (during 5–7 weeks).	Explicit instruction about cue-reminder aim.Duration of exposure: 5–7 weeks	+0.46 (−0.44 to 1.36), *p* = 0.32
Lange (17)	Objectively assessed alcohol consumption (breath alcohol concentration, abstinence of alcohol)	1,412/376 groups	20.37 (2.75)	C1: intervention only (*n* = 55)C2: intervention plus cue-reminder (*n* = 57)	T1: hours after intervention (and after cue-reminder exposure in C2)	Groups were asked who the designated drivers was for the evening.	Wearing of a bracelet with the printed words “designated driver” until post-test	Explicit instruction about cue-reminder aims.Duration of exposure: a varying number of hours	+0.23 (−0.15 to 0.61), *p* = 0.23
Hermans unpublished (18)	Objectively assessed alcohol consumption (alcohol vs. no alcohol consumed, number of ml consumed alcoholic beverage)	207	20.50 (1.87)	C1: intervention only (*n* = 103)C2: intervention plus cue-reminder (*n* = 103)	T1: directly after intervention (and after cue-reminder exposure in C2)	Exposure to a film about environmental pressure and alcohol use. It focused on social norms, pressure to drink, external influences of commercials, and happy hours and refusal skills. A symbol resembling a power button was embedded in the film.	Exposure to double-sided coasters with pictures of the symbol (power button) in a bar. The coasters were added to 30-min visit to an experimental drinking situation where they were distributed on all the tables in the bar	No explicit instruction about cue-reminder aim.Duration of exposure: 30 min	−0.35 (−0.62 to −0.08), *p* = 0.01

In all studies participants were randomized to an experimental group receiving an intervention including a cue-reminder or a control condition receiving the intervention only. One study had a pretest–posttest design (14), whereas the other studies had a posttest only design. In three studies, participants were exposed to the cue-reminder in a test session after the intervention (15, 16, 18), whereas in the other three studies, exposure to the cue-reminder took place in participants' daily lives (4, 14, 17).

All studies included participants of both sexes. Three studies included more females than males in their design (4, 16, 18), one study included more males than females (15), and two studies included an almost equal number of both males and females (14, 17). In terms of sample age, one study included 8 year-old participants (15), four studies included participants with a mean age of ~20 years (4, 16–18), and one study included participants with a mean age of 37 years (14). The majority of studies sampled highly educated participants (4, 14, 16, 18). One study did not report participants' educational level (17) and one study focused exclusively on children in elementary school (15). Finally, four studies included between 100 and 200 participants in their design (4, 15, 16, 18), whereas two studies included a substantial larger number of participants with 1,412 and 2,634 participants, respectively (14, 17).

### Systematic Review

In addition to variation in participants' characteristics, we observed a variation in how the cue-reminders were applied in the included studies. First, the studies differed in whether or not the aim of the cue-reminder was made explicit to intervention recipients. Second, the studies differed in the duration to which intervention recipients were exposed to the cue-reminder.

#### Explicitness of Cue-Reminder Aims

In three studies, participants were explicitly told that the aim of the cue-reminder was to remind them of intervention-related information (4, 14, 17). Thereby, the interventionists attempted to create an explicit association between the cue-reminder and the intervention message. In the study of Dal Cin et al. (4), participants were given a bracelet and were explicitly told to think about the dangers of unsafe sex whenever they looked at the bracelet. Likewise, Lange et al. (17) asked designated drivers to wear a bracelet with the words “designated driver” printed on it. Their goal was to increase the activation and accessibility of the designated driver concept in memory for those who reported being the designated driver. Finally, in the study of van Lettow et al. (14) participants were offered a bracelet or asked to self-select an object of frequent use and were instructed to think of their alcohol consumption-related action plans whenever they looked at their bracelet or self-selected object.

In contrast, in the other three studies (15, 16, 18), participants were not specifically told about the presence of a cue-reminder nor about the aim of the cue-reminder. Thereby, no explicit association was attempted between the cue-reminder and the intervention message. In Bevelander et al. study (15) children in the control condition received a short interactive lesson featuring photos, video clips, and real-life situations, and were taught about social modeling effects on their food intake. In the experimental condition, the experimenter introduced a monkey puppet at the start of the intervention and used this puppet to explain social modeling and communicate the prevention message. In a subsequent test session, the monkey-puppet was merely present. Kleinjan et al. (16) and Hermans et al. (18) also did not explicitly instruct participants about the cue-reminders. In both studies the cue-reminder was a visual symbol (“The Power Button”) that was embedded in an educational prevention film. The aim of the film was to educate participants about environmental pressure and alcohol use. This symbol was also printed on the drink coasters that were present in a subsequent test session in a bar. By doing so, the authors hoped that the cue-reminder would implicitly trigger the recall of the intervention message.

#### Duration of Exposure to the Cue-Reminder

Included studies also differed in the extent to which participants were exposed to the cue-reminder after the intervention. In four studies, participants were exposed to the cue-reminder for a relatively short period of time. Bevelander et al. (15) exposed children to the cue-reminder (i.e., the monkey puppet) during a 10-min test session in which they were allowed to eat candy. Hermans et al. (18) and Kleinjan et al. (16) exposed their participants to their cue-reminder for 30 and 45 min, respectively. Finally, Lange et al. (17) asked designated drivers to wear the bracelet for a couple of hours until their return of their night out. In two studies, however, participants were exposed to the cue-reminder for a relatively longer period of time. In the study of van Lettow et al. (14) participants were requested to use the cue-reminder for 1 month, whereas Dal Cin et al. (4) asked their participants to wear the bracelet at all times until the post-intervention measurement 5–7 weeks later.

#### Effect Sizes

Table 2 presents the effect sizes of the cue-reminders per study. Because no data on the long-term effects of cue-reminders on health-risk behaviors were available, we only present the effect sizes of the cue-reminders on the behavioral outcome(s) measured directly after the exposure period. From the six studies, only one study demonstrated a positive and significant small effect (14). Specifically, this study demonstrated that the intervention with cue-reminder was more effective in reducing alcohol consumption than the intervention only. In another study, a negative and significant effect size was found (18). In the group receiving the intervention with cue-reminder, alcohol consumption was higher as compared to the group receiving the intervention alone. In the remaining four studies, the effect sizes were positive but not significant.

### Risk of Bias

Of the seven assessment items, the number of items judged as unclear risk of bias per ranged from 0 to 3 per study (*M* = 2.33, *SD* = 1.21). The number of items judged as low risk of bias ranged from 2 to 5 per study (*M* = 3.33, SD = 1.03). The number of items judged as high risk of bias ranged from 0 to 3 per study (*M* = 1.33, SD = 1.03). In four studies high risk of bias was expected due to blinding of outcome assessment. Two studies relied on participants' self-reports of health-risk behavior, along with participants' awareness of exposure to a cue-reminder (4, 14). Two other studies used more objective outcome assessment methods, but the assessors were not blinded for condition (16, 18). In all but one study (14), risk of bias in relation to random sequence generation and allocation concealment was not reported and therefore judged as unclear risk of bias.

## Discussion

The aim of this systematic review was to explore whether there is evidence supporting the proposition that cue-reminders increase the effectiveness of health-risk behavior interventions. Therefore, we examined the added effect of complementing interventions with cue-reminders. In addition, we explored how cue-reminders are applied in health-risk behavior interventions to date.

Six studies were included in this systematic review. Four studies focused on the reduction of alcohol consumption, one study focused on prevention of unhealthy dietary intake, and one study focused on prevention of unsafe sex. Two studies demonstrated significant but contrasting effects of the cue-reminder on participants' health-risk behavior, as measured directly after exposure to the cue-reminder. One study (14) demonstrated a positive, small effect of the cue-reminder: exposure to the cue-reminder was significantly and positively associated with reduced alcohol consumption. In contrast, another study (18) demonstrated a negative, medium effect of the cue-reminder: exposure to the cue-reminder increased participants' alcohol consumption. In the remaining four studies, the effect sizes were positive but not significant. Thus, evidence in support of the proposition that cue-reminders increase the effectiveness of health risk-behavior interventions was found in one study.

A qualitative examination of the included studies revealed that the studies differ on multiple aspects: (1) characteristics of the study sample, (2) whether the design was a pretest—posttest design vs. posttest only design, (3) whether exposure to the cue-reminder took place in participants' daily lives vs. during a test session, (4) whether participants were explicitly instructed about the aim of the cue-reminder during the intervention, and (5) the extent to which participants were exposed to the cue-reminder after the intervention. Although these differences increase our understanding of the potential ways cue-reminders can be applied, these differences complicate the comparability of the studies and thus the identification of the specific factor(s) responsible for the varying findings related to cue-reminder effectiveness.

Future research may therefore manipulate specific factors that are expected to influence cue-reminder effectiveness. It is possible, for instance, that providing an explicit instruction about the cue-reminder may contribute to the effectiveness of the cue-reminder. When the aim of the cue-reminder is made explicit, the likelihood that intervention recipients associate the cue-reminder with the intervention message may be higher than when the aim is not made explicit. As a consequence, observing the cue-reminder may lead to a better retrieval of relevant health information associated with the intervention. Further, one may better remember one's healthy intentions. Also, when intervention recipients are instructed to expose themselves to the cue-reminder, it is possible that this might to lead higher commitment to the intervention aims. That is, by making such a commitment, it is possible that they feel more pressure to behave consistent with the intervention message and are more likely to reach the behavior goals that are linked to the intervention (19). To increase our understanding of the effectiveness of cue-reminders as well as whether explicit instructions contribute to its effectiveness, future studies might compare the effects of an experimental condition that includes a cue-reminder and gives explicit instructions about the cue-reminder aims with an experimental condition in which the cue reminder is not made explicit or in which no cue reminder is present at all. Similarly, it may be expected that exposure duration could affect cue-reminder effectiveness. That is, longer exposure periods may provide more opportunities for recipients to retrieve relevant health information and act upon their positive intentions, resulting in stronger behavior change effects. On the other hand, longer exposure periods may be less impactful because of reduced sensitivity to the cue-reminder due to its longer presence. To elucidate whether and how the length of exposure period influences the ability of cue-reminders to increase intervention effectiveness, future studies may involve multiple cue-reminder conditions, varying in the duration of exposure to the cue-reminder after the intervention.

The studies included in this systematic review describe potential psychological mechanisms that may be involved in cue-reminder effectiveness, but these are yet to be explored. For example, multiple studies recognize that health-risk behaviors are automatic or impulsive, triggered by contextual cues, such as observing or feeling pressure from peers who are drinking alcohol or eating candy (14–16). The salience of the cue-reminder in such risk-behavior inducing contexts may then serve as an inhibiting cue, disrupting the health-risk behavior. This disruption may be due to enhanced awareness or increased activation and accessibility of the intervention message in memory of the intervention message (14, 16, 17) or the recall of one's personal goals (14). Dal Cin et al. (4) adds that cue-reminders are expected to increase the personal relevance of intervention messages. While participating in an intervention, recipients may not see the importance of the intervention message for their own lives. Cue-reminders may increase the salience of the message at the time and place the behavior is occurring, making the message more personally relevant. To gain insight into how cue-reminders may increase the effectiveness of health-risk behavior interventions, a suggestion for future research is to focus on the psychological processes that may be involved in cue-reminder effectiveness.

### Strengths and Limitations

The present systematic review is the first systematic evaluation of the added effect of cue-reminders in health-risk behavior interventions, which can be considered a strength. Nonetheless, this review is also subject to limitations. Firstly, despite a broad search strategy for both published and unpublished studies and the retrieval of many potentially relevant records, only six studies matched the inclusion criteria. Although the effectiveness of health-risk behavior interventions with cue-reminders was explored in multiple studies, many studies involved an experimental group which received an intervention including a cue-reminder and a control receiving no intervention. With such a study design, the independent effect of the cue-reminder cannot be established. Secondly, given the limited number of studies and heterogeneity across studies, we were not able to perform a meta-analysis to quantitatively explore whether cue-reminders increase the effectiveness of health-risk behavior interventions. A meta-analysis of few studies is only informative if the studies are highly similar (20), which was not the case with the studies included in this review. To provide as much insight as possible into whether complementing interventions with cue-reminders increases the effectiveness of these interventions, we therefore reported the effect sizes and their confidence intervals for each individual study [cf., (20)]. Thirdly, all included studies assessed only the immediate effects of exposure to cue-reminders on the health-risk behavior of interest. Therefore, our exploration of whether cue-reminders increase intervention effectiveness is also limited to short-term effectiveness. To acquire more insight into the potential long-term effects, future studies should include a follow-up measurement.

### Practical Implications

The results of the present systematic review are of value for intervention developers who are planning to complement their health-risk behavior interventions with cue-reminders. The varying and contrasting effect sizes show that effectively complementing interventions with cue-reminders may be a challenge. Although cue-reminders may be perceived as a simple add-on intervention component to increase intervention effectiveness, this review shows that intervention developers have multiple points to consider.

## Conclusion

To date, only six studies have been performed to investigate whether adding cue-reminders to interventions increases the effectiveness of health-risk behavior interventions. Because of the heterogeneity across the studies in terms of sample, research design, and how the cue-reminder is applied, it remains largely unclear whether and how cue-reminders increase the effectiveness of health-risk behavior interventions. Nevertheless, this systematic review provides a valuable insight into which practical issues have to be considered by intervention developers who are planning to complement their health-risk behavior interventions with cue-reminders.

## Data Availability

The datasets generated for this study are available on request to the corresponding author.

## Author Contributions

LvL, MK, and RH were involved with conceptualization of the research, data interpretation, and writing up the manuscript. BvdP was involved with conceptualization of the research and data interpretation. LvL and SO were involved with analyses and data interpretation. LvL, SO, BvdP, LL, RCME, MK, and RCJH contributed to manuscript revision, read, and approved the submitted version.

### Conflict of Interest Statement

The authors declare that the research was conducted in the absence of any commercial or financial relationships that could be construed as a potential conflict of interest.
